# A Vote for School Lunches: School Lunches Provide Superior Nutrient Quality than Lunches Obtained from Other Sources in a Nationally Representative Sample of US Children

**DOI:** 10.3390/nu9090924

**Published:** 2017-08-24

**Authors:** Jacqueline A. Vernarelli, Brady O’Brien

**Affiliations:** Department of Biology, Fairfield University, Fairfield, CT 06468, USA; bobrien@naviencesystems.com

**Keywords:** obesity, school lunch, NHANES, energy density, diet quality

## Abstract

Childhood obesity is an ongoing public health program. As such, a major public health research objective is to identify potential targets for intervention; one such area is school lunches (SL). The National School Lunch Program (NSLP) serves over 31 million children each day; the National Health and Nutrition Examination Survey (NHANES) is uniquely positioned to allow researchers to assess diet quality in federal nutrition assistance programs. The objective of the study was to investigate whether lunches provided by schools provide different nutritional value than lunches obtained elsewhere. In a nationally representative sample of 2190 children, consumption of a school-provided lunch (SL) was associated with greater nutritional quality compared to lunches obtained elsewhere across both age and income categories. Children who were eligible for no-cost school lunch, but did not participate in the NSLP consumed approximately 60% more energy, 58% more total fat, 60% more saturated fat, 50% more solid fat, 61% more sodium, double the amount of added sugars and less than half the amount of fruit than NSLP participants (all *p* < 0.001). The results of this study suggest that though widely criticized, school lunches provide superior nutrient quality than lunches obtained from other sources, particularly for low-income children.

## 1. Introduction

Childhood obesity rates have increased rapidly in the past 40 years, with 17% of US children age 2–19 classified as obese in the most recent population data [1]. As such, a major public health research objective is to examine nutritional intake among US children as a strategy of identifying potential targets for intervention; one such area is school lunches (SL). The National School Lunch Program (NSLP) serves over 31 million children each day, making school lunch a significant contributor to dietary intake among school age children [2]. In 2008, the Institute of Medicine (IOM) examined the nutritional content of school lunches, concluding in a report that school lunches provided too few servings of fruits and vegetables, and too many servings of refined grains and saturated fats. The IOM report led to a revision of NSLP guidelines, Healthy, Hunger-Free Kids Act (HHFKA) passed in 2010 [3,4] that would revise school meal standards to be more in line with current public health objectives for obesity prevention and nutrient intake, including the most recent version of the *USDA Dietary Guidelines for Americans* (DGA) beginning in 2012. Adequacy in nutrient intake among school children is also a public health concern with *Healthy People 2010* goals including increasing intake of fruits and vegetables, decreasing intake of sodium and saturated fats; reducing the number of calories from solid fats and added sugars has also been listed as a proposed goal for Healthy People 2020 [5,6]. In a recent report issued by the USDA, all school children fell far short of the Dietary Guidelines for Americans, with an average Healthy Eating Index (HEI)-2005 score of only 58 [7]. A 2010 USDA Economic Research Report evaluated the impacts of foods consumed away from home, including school lunches, on diet quality in children. The report identified away from home eating, including consumption of school lunch, was associated with greater energy intake and lower diet quality as measured by the 2005 HEI [8]. Additional national studies have affirmed the USDA report, indicating that that meals served for school lunch are lacking in nutrient quality—specifically when it comes to sodium, fat, and fiber [9,10]. However, these publications often exclusively examine only foods consumed at school, or meals consumed away from home.

Relatively few published articles have compared nutrient intake among students with lunches obtained in the school cafeteria with lunches brought from home or obtained elsewhere [11]. One single-school study of second-grade students found that meals packed from home contained fewer fruits and vegetables, and more salty grain snacks than meals served in the school cafeteria. Another group of researchers examined only the nutritional content of lunches brought from home in a single Texas school district, and compared these lunches with NSLP guidelines [11]. The authors found that lunches brought from home did not meet NSLP standards, specifically exceeding recommendations for sodium, and not meeting recommendations for fruit, and suggested that intervention is needed to improve lunch quality of meals brought from home [11]. These studies have been conducted in single districts, or even single schools, therefore limiting the conclusions that can be drawn regarding a large national school lunch program. Therefore, the objectives of the present study were to assess the nutrition content of school children’s lunches obtained from a school cafeteria compared with lunches brought from home or obtained elsewhere in a nationally representative sample of school-aged children made possible by analysis of data from the National Health and Nutrition Examination Survey [12]. Difference in meal nutrient quality and energy content based on age group (4–8 years vs. 9–13 years vs. ≥14) and school socioeconomic status (based on eligibility rates for free or reduced-price (FRP) meals) were also explored.

## 2. Materials and Methods

This study examined the quality of lunches consumed by a nationally representative sample of school children that participated in the 2009–2012 National Health and Nutrition Examination Survey (NHANES). The NHANES is a large, cross-sectional survey conducted by the National Center for Health Statistics. NHANES and its related nutritional component *What We Eat In America* (WWEIA) are designed to monitor the health and nutritional status of non-institutionalized civilians in the US. Nationally representative survey and physical data are collected on a continual basis and released in two-year increments. Complete details regarding the NHANES sampling methodology, data collection, and interview process are available on the NHANES website; written consent was obtained from all subjects [13]. For the present study, school children were identified as NHANES participants who affirmed attending school (kindergarten through high school) and reported consuming a lunch meal at school (*n* = 2190). Of this eligible sample, there was an equal proportion of children who reported that they obtained their lunch meal from the school cafeteria1 (*n* = 1108; categorized as school-provided lunch, SL consumers) vs. those who obtained their meal from elsewhere (*n* = 1082; categorized as “non-consumers”); these non-cafeteria sources include: lunches brought from home, obtaining food from someone else, buying food outside of school grounds and eating on the premises, and obtaining foods from a vending machine. There are over twenty possible options for the source of an individual food/beverage. To focus specifically on school-provided lunches, further categorization of food source was not done. Children who attended school with a universal school lunch policy were not identified in the dataset. In order to analyze age differences, children were categorized into the following age groups: 4–8 years, 9–13 years, and ≥14 years. A second set of analysis was completed in order to evaluate intake among children of varying socioeconomic status by identifying children who participate in Free or Reduced Price (FRP) lunch programs, i.e. children categorized as paying no cost, reduced cost, or full-price for their school lunch. These classifications allowed comparing children who participate in the FRP school lunch program with income-eligible non-participants identified using NSLP Income Eligibility Guidelines.

### 2.1. Dietary Intake

Children who participated in NHANES provided one day of dietary recall data including all foods and beverages during their visit to the mobile examination unit as part of the *What We Eat in America Study* [14]. A single day of recall is used to monitor the dietary behaviors of the US population, and has been established as a way of assessing the mean of the population′s usual dietary intake. Dietary recall data were collected in-person by trained interviewers using the automated multi-pass method of 24-h recall, accounting for sample variability and intake day of week. Specific status codes were provided in the NHANES dataset to indicate the quality, reliability, and completeness of the dietary data.

The USDA Food and Nutrient Database for Dietary Studies, version 5.0 and FNDDS 2011–2012 were used to process NHANES dietary data from the 2009–2010 and 2011–2012 NHANES survey cycles, respectively, as well as the 2011–2012 and 2009–2010 USDA Food Patterns Equivalents Database (FPED) [15]. The FPED converts ≥7000 individually reported food and beverage items into 37 disaggregated USDA Food Patterns components (e.g., added sugars, solid fats, total fruit, dark vegetables, etc.) allowing for comparison to recommendations of the Dietary Guidelines, as well as NSLP standards and *Healthy People* guidelines. In addition to food pattern components, dietary energy density (ED) was also calculated for each individual. While there are several methods to calculate dietary ED, there is no standardized method for selecting which foods and/or beverages to include in the calculation. Current literature has suggested that using a food-only method for calculating energy density may provide the most robust results, since beverages can contribute disproportionately to overall dietary ED due to their high gram weight and high water content [16,17] and can mask relationships between foods in the diet and markers of disease [17]. In preliminary analyses, dietary ED was calculated two ways: using all foods and beverages, and using foods only. For each method, ED was calculated dividing the energy content (kcal) by weight of foods or beverages (g) consumed. USDA food codes were used to identify which items were foods, and which were beverages (e.g., differentiating between milk used in cereal vs. milk consumed as a beverage). During these initial analyses, the disproportionate contribution of beverages to overall ED was observed and further analyses were conducted using the food-only method, controlling for beverage ED; more importantly, since milk served at a school cafeteria can widely vary based on student age, school district, and preferences, beverages were eliminated in order to analyze the data looking at only the foods reported. Overall dietary ED and meal ED were calculated for each individual by totaling the food-only energy intake (kcal) and dividing by the total gram weight of foods consumed. For ease of discussion, children who responded that they did not consume a school-provided lunch will be referred to as “non-consumers”.

### 2.2. Statistical Analysis

For the present analyses, we included all children age 4–19 who attended kindergarten through high school and reported consuming a lunch meal. Age at the time of exam, sex, and race/ethnicity and socioeconomic status were all provided in the NHANES dataset. Age- and sex-specific body mass index (BMI) percentiles were calculated from the dataset using a SAS program provided by the CDC (http://www.cdc.gov/nccdphp/dnpao/growthcharts/resources/sas.htm) to correspond with data collected by the NHANES. Socioeconomic status was quantified as a continuous variable using poverty-income ratio (PIR), or the ratio of family income to family-size specific poverty threshold, and was used to determine income eligibility for FRP lunches using the NSLP Income Eligibility Guidelines.

All data were analyzed using SAS version 9.3 (SAS Institute, Cary, NC, USA). Specific survey procedures were used in the analysis to account for sample weights, unequal selection probability, and clustered design. Initial comparison of mean energy intake of each body weight group (lean, overweight, and obese) indicated that no statistical difference in energy intake exists, making an adjustment for energy intake unnecessary. Multivariate regression models were then used to evaluate the relationship between lunch source (school cafeteria vs. non-cafeteria) and nutritional intake (energy, macro- and micronutrients, and food patterns equivalents). Age-group regression models were adjusted for age in years, race, sex and socioeconomic status (PIR). Income-category models were also energy-adjusted with significance determined at *p* < 0.05.

## 3. Results

In this nationally representative sample of 2190 children, roughly half of the weighted sample were female (51.4%), with the majority of children identifying as non-Hispanic white (56.5%), and approximately half of the children were income eligible for free or reduced price (FRP) lunch (37.2% PIR < 130%; 12.8% with PIR 130–185%). There was an even distribution of reported consumption of school-provided lunches (SL consumers; 50% weighted sample) and lunches obtained elsewhere (Non-consumers, 50% weighted sample) for the specific 24-h recall data used in this study, however, 84% of the children responded that they regularly consume school lunch at least one day each week. These, and other demographic characteristics are presented in Table 1. A subset of these children (*n* = 1809) provided data regarding school lunch program participation; in this sample, 50% of the children qualified for no-cost lunches, 8% for reduced-price lunches, and 42% were not income eligible for FRP lunches.

### 3.1. Dietary Intake by Age Group

In young children (age 4–8 years), SL consumers had significantly lower meal energy density, fewer grams of carbohydrates, fewer grams of total fat and fewer grams of saturated fat and fewer teaspoons of added sugars and consumed 20% fewer calories than non-consumers. No differences in sodium intake or other food pattern components were observed for this age group. In children age 9–13 years SL consumers reported 25% less energy, as well as significantly lower meal energy density, fewer carbohydrates, less total and saturated fat, as well as less sodium and added sugars than non-consumers. In this age group, SL consumers also had lower intakes of both total and whole grains, and fewer servings of protein foods than non-consumers. In children over 14 years, the only observed difference between SL consumers and non-consumers was meal energy density, with school-provided lunches significantly lower in energy density than lunches obtained elsewhere (2.19 vs. 2.51 kcal/g, *p* = 0.01). It is notable that school-provided lunches and lunches obtained elsewhere did not differ in servings of vegetables, fruits, or refined grains in any age group. Though total vegetable consumption did not differ between SL consumers and non-consumers between the ages of 4–13, difference in intake for various vegetable subtypes did exist. Across all age categories, SL consumers report fewer servings of starchy vegetables than non-consumers (4–8 years 0.07 vs. 0.12 cup equiv., *p* = 0.03, 9–13 years 0.03 vs. 0.13 cup equiv., *p* < 0.0001; ≥14 years 0.08 vs. 0.17 cup equiv., *p* = 0.002). Reported intake levels of dark green vegetables, red and orange vegetables and legumes counted as vegetables, as well as intakes of fruit subtypes (citrus, melon, berry, and juice) were not statistically significant (Table 2).

### 3.2. Dietary Intake by Socioeconomic Status

Dietary intake was also evaluated by socioeconomic status groups. This allows a comparison of the nutrient intakes of NSLP participants receiving FRP lunches with income-eligible non-participants. In students who were not eligible for FRP lunches, only two differences in intake patterns were seen, with non-consumers having greater intake of both whole grains and vegetables than SL consumers (Table 3). No differences in nutritional intake were observed among students eligible for reduced price (but not free) school lunches. There were, however, significant differences in nutritional intake among students who were eligible for no-cost lunches. Income-eligible NSLP non-participants consumed approximately 60% more energy, 66% more carbohydrates, 58% more total fat, 60% more saturated fat, 50% more solid fat, and less than half the amount of fruit than NSLP participants. These provided lunches also had 1.6 times the amount of sodium and over double the amount of added sugars than school-provided lunches.

### 3.3. Body Measures

Evaluation of differences in total daily energy intake, lunch energy intake, and BMI-for-age percentile indicated that there were no differences in BMI percentile between SL consumers and Non-consumers (68.9 vs. 63.1, *p* = 0.08) and Non-consumers consumed an average of 100 kcal/day more than SL consumers. No difference in BMI percentile was observed when comparing socioeconomic groups of (no-cost lunch 69.5, reduced price 68.4, and full-price 63.5).

## 4. Discussion

### 4.1. Quality of School-Provided Lunches Compared to Lunches Obtained Elsewhere by Age Group

The results from this study indicate that school-provided lunches provide better nutrition than lunches obtained elsewhere, but that the nutritional comparison of school-provided lunches with lunches obtained elsewhere differed by age group. On average, across all age groups, school-provided lunches are lower in energy density, total fat, saturated and added-sugars than lunches obtained elsewhere. For children age 4–13 years, school lunches also were lower in energy content, carbohydrate content, and whole grains. In children age 9–13, school lunches were also lower in sodium, and protein foods. Little difference between school-provided lunches and lunches obtained elsewhere for older children (>14 years), possibly because of the variation in available food selection served at high schools. This finding is consistent with the findings of Caruso and colleagues who evaluated nutrition content of lunches brought from home by elementary and intermediate school children [11]. The findings in the present study are contradictory to earlier work done by Briefel and colleagues, who compared intake patterns among school aged children who reported consuming foods at home, at school, and from other sources, and determined that NSLP participants consumer greater quantities of energy-dense foods than children who do not participate in the NSLP during the school day, but not outside of school [18]. The authors suggest that nutrient intake of school students may be improved by allowing reducing the frequency that fried potatoes and sweetened baked goods are served at schools [18]. The present analysis has found that competitive foods may not be the solution, as NSLP participants have significantly lower meal energy density than non-NSLP participants.

### 4.2. Quality of School-Provided Lunches vs. Lunches Obtained Elsewhere by Socioeconomic Status

The greatest differences between nutritional content of school-provided lunches and lunches obtained elsewhere was seen when stratifying children by FRP eligibility. Among children who are eligible to receive lunch at no-cost, school provided lunches provide significantly better nutritional value than lunches obtained elsewhere. To date, there are no published articles evaluating the differences in nutritional intake between NSLP participants and income-eligible non-participants. Although the literature is sparse, this study provides important information regarding potential interventions that can increase participation in FRP meal programs.

This study has several limitations. Even though the NHANES data are nationally representative, they are collected cross-sectionally and the present study uses only a single 24-h recall for assessment of nutrient intake. Future cohort studies may be conducted to address intake over an extended period of time. Even though dietary data were collected using the best available tools, dietary data for children under age 6 were obtained via proxy (whomever best knows the child); dietary data for children age 6–8 were obtained by interviewing a proxy with the child present for assistance; and data for children 9–12 were obtained by interviewing the child with the assistance of a proxy, with may limit the accuracy of data collected. In addition, children who attend a school with a universal school lunch policy are not specifically identified in the NHANES dataset. In addition, this study uses data that were collected prior to the implementation of the Healthy Hunger-Free Kids Act. It is unclear whether implementation of this Act will have any impact of the trends in intake.

## 5. Conclusions

The results of this study suggest that, although widely criticized, school lunches provide superior nutrient quality than lunches obtained from other sources, particularly for low-income children. Interventions and public health messaging should focus on increasing enrollment in the NSLP, and educating parents on the quality of meals provided by schools as opposed to meals brought from home.

Results from this study indicate that NSLP participation provides students with meals that are, on average, lower in energy density and more favorable in macronutrient and food-group composition than meals brought from home or obtained elsewhere. In addition, in low-income children who qualify for lunches provided free of charge, NSLP lunches obtained from the school cafeteria are of significantly higher nutritional value than lunches obtained elsewhere by children who are income-eligible for free lunch but choose not to participate in the NSLP. Public health messaging should promote NSLP participation, particularly among low-income families. Although more investigation is needed using newer data, these results indicate that participation in the NSLP among low-income children increases intake of foods that are low in energy density, essential food groups (such as fruits) while limiting intake of sodium, added sugars, solid fats, saturated fats, refined grains, and energy.

## Figures and Tables

**Table 1:** Demographic Characteristics of NHANES 2009–2012 Eligible School Children.

	*n*	Weighted *n*	%
Sex			
Female	1072	14,068,660	51.4
Male	1118	13,309,781	48.6
Age Category			
4–8 years	786	8,982,318	35.1
9–13 years	825	9,824,053	38.5
≥14 years	452	6,763,056	26.4
Race			
Non-Hispanic White	615	15,470,648	56.5
African American	579	3,993,140	14.6
Mexican-American	484	3,724,629	13.6
Other	512	4,190,024	15.3
Poverty-Income Ratio			
<130%	1043	10,191,928	37.2
130–185%	293	3,507,160	12.8
>185%	854	13,679,354	50.0
Lunch Source			
Cafeteria at School	1108	13,699,939	50.0
Other	1082	13,678,503	50.0
Regularly Consume School Lunch			
No	279	4,172,193	15.2
Yes	1911	23,206,249	84.8
School Lunch Program Participation Subset—by Income			
Lunch Price			
Free/No cost	1128	10,727,709	50.1
Reduced Price	168	1,708,276	8.0
Full price	513	8,993,561	41.9

Note: Eligibility sample were identified as currently attending school (kindergarten through 12th grade); reported consuming lunch; lunch was consumed away from home.

**Table 2:** Nutritional Intake of Students Consuming School-Provided Lunch (SL) vs. Lunch Obtained Elsewhere (Other lunch) by Age Group.

Age Group	4–8 Years		9–13 Years		≥14	
	Adj. Mean	SE	Adj. Mean	SE	Adj. Mean	SE
Macronutrient Content						
Lunch Energy Content (kcal)						
School lunch	369.9	15.3	410.8	18.2	560.2	47.7
Other lunch	459.2	17.8	544.7	23.7	630.3	47.7
*p*-value		0.0013		<0.0001		0.41
Meal Portion (grams)						
School lunch	246.6	11.7	252	34	240.4	9.3
Other lunch	298.3	8.26	314.32	35.4	269.7	8.7
*p*-value		0.006		0.0016		0.07
Meal Energy Density (kcal/g)						
School lunch	1.97	0.07	2.04	0.08	2.19	0.1
Other lunch	2.31	0.08	2.35	0.12	2.51	0.09
*p*-value		0.0055		0.007		0.01
Meal carbohydrate s (g)						
School lunch	44.0	2.1	47.0	1.8	58.0	4.1
Other lunch	54.0	2.1	62.7	2.7	65.8	4.8
*p*-value		<0.0001		0.6		0.11
Meal protein (g)						
School lunch	15.7	0.8	18.4	0.9	25.4	2.9
Other lunch	17.2	0.8	21.7	1.0	26.0	2.5
*p*-value		0.002		<0.0001		0.30
Meal Total Fat (g)						
School lunch	14.9	0.6	16.9	1	25.4	2.7
Other lunch	19.9	1.1	23.5	1.3	29.6	2.7
*p*-value		0.0004		0.0003		0.37
Saturated Fat (g)						
School lunch	4.9	0.3	5.6	0.3	9.0	1.2
Other lunch	6.4	0.4	7.1	0.4	8.2	1.0
*p*-value		0.0075		0.0068		0.7
Lunch Food Pattern Equivalents and Other Nutrients of Concern						
Sodium (mg)						
School lunch	771.7	36.8	825.7	48.7	1348.2	172.8
Other lunch	874.5	44.8	1094.6	51.0	1278.2	148.8
*p*-value		0.11		0.0004		0.81
Dietary fiber (g)						
School lunch	3.7	0.2	3.7	0.2	4.3	0.3
Other lunch	4.1	0.2	4.9	0.3	4.9	0.4
*p*-value		0.21		0.0011		0.20
Added sugars (tsp. equiv.) ^2^						
School lunch	1.2	0.1	1.5	0.1	1.9	0.5
Other lunch	2.2	0.2	2.6	0.3	2.4	0.6
*p*-value		<0.0001		0.003		0.64
Meal solid fats (g equiv.)						
School lunch	8.0	0.6	8.5	0.5	14.2	2.5
Other lunch	8.9	0.9	9.9	0.8	11.0	1.9
*p*-value		0.51		0.13		0.42
Vegetables (cup equiv.) ^3^						
School lunch	0.26	0.02	0.29	0.04	0.34	0.05
Other lunch	0.30	0.03	0.36	0.04	0.54	0.05
*p*-value		0.18		0.26		0.007
Fruits (cup equiv.) ^4^						
School lunch	0.33	0.03	0.31	0.04	0.29	0.08
Other lunch	0.28	0.04	0.22	0.04	0.13	0.07
*p*-value		0.26		0.09		0.20
Grains (ounce equiv.)						
School lunch	1.7	0.1	2.0	0.1	2.6	0.3
Other lunch	2.0	0.1	2.4	0.1	2.7	0.3
*p*-value		0.27		0.017		0.80
Whole Grains (ounce equiv.)						
School lunch	0.05	0.02	0.11	0.02	0.13	0.03
Other lunch	0.25	0.04	0.23	0.04	0.18	0.04
*p*-value		<0.0001		0.011		0.2
Refined Grains (ounce equiv.)						
School lunch	1.69	0.14	1.89	0.1	2.44	0.27
Other lunch	1.72	0.11	2.15	0.14	2.53	0.26
*p*-value		0.002		0.385		0.63
Protein Foods (ounce equiv.) ^5^						
School lunch	1.1	0.1	1.2	0.1	1.8	0.4
Other lunch	1.3	0.1	1.7	0.1	2.1	0.4
*p*-value		0.19		0.014		0.64

Note: Regression models with adjusted (least-squared) means presented, representing population intake during the lunch meal. Models are adjusted for age in years, sex, race, and income, as quantified by Poverty-Income Ratio based on household size and survey cycle year. ^2^ Added sugars is calculated using sugar content of foods and ingredients defined by the USDA as added sugars; ^3^ Vegetables includes dark green, red, orange, starchy and other vegetables; ^4^ Fruits includes total intact fruit (whole or cut) and fruit juices used as sweeteners in foods, but excludes fruit juices consumed as beverages; ^5^ Protein foods includes meat, poultry, organ meat, cured meat, fish, eggs, nuts, seeds, and soy products.

**Table 3:** Nutritional Intake of Students Consuming School-Provided Lunch (SL) vs. Lunch Obtained Elsewhere (Non-SL) by Socioeconomic Status (SES).

Family SES	<130% PIR ^1^		130–185% PIR		>185% PIR	
	Eligible for Free/No-Cost Lunch		Eligible for Reduced Price Lunch		Full Price Lunch	
	Mean	SE	Mean	SE	Mean	SE
Lunch Energy Content (kcal)						
School lunch	414.3	17.2	393.5	22.9	493.2	31.6
Other lunch	654.0	32.9	458.2	43.3	539.5	36.6
*p*-value		<0.0001		0.20		0.27
Meal Portion (grams)						
School lunch	234.6	8.6	232.0	16.5	234.9	15.8
Other lunch	309.3	16.4	213.9	20.6	244.5	20.7
*p*-value		<0.0001		*0.40*		0.72
Meal Energy Density (kcal/g)						
School lunch	1.90	0.03	1.95	0.09	2.14	0.07
Other lunch	2.11	0.04	2.02	0.11	2.18	0.07
*p*-value		<.0001		*0.65*		0.58
Meal carbohydrate s (g)						
School lunch	47.7	2.0	50.0	3.1	55.6	3.5
Other lunch	71.0	4.3	52.7	5.3	63.2	4.4
*p*-value		<0.0001		0.60		0.11
Meal protein (g)						
School lunch	18.2	1.0	15.4	1.1	21.4	1.9
Other lunch	26.9	1.5	17.8	2.2	19.9	2.0
*p*-value		<0.0001		0.39		0.60
Meal Total Fat (g)						
School lunch	17.1	0.9	15.2	1.0	20.8	1.6
Other lunch	29.5	1.6	19.7	2.0	23.7	2.0
*p*-value		<0.0001		0.06		0.20
Saturated Fat (g)						
School lunch	5.5	0.3	5.3	0.4	7.0	0.7
Other lunch	9.2	0.5	6.2	0.7	6.7	0.7
*p*-value		<0.0001		0.38		0.74
Lunch Food Pattern Equivalents and Other Nutrients of Concern						
Sodium (mg)						
School lunch	843.9	36.2	848.3	80.3	1010.1	88.9
Other lunch	1333.7	73.1	902.0	114.1	983.6	122.0
*p*-value		<0.0001		0.67		0.87
Dietary fiber (g)						
School lunch	3.9	0.2	4.0	0.4	3.9	0.3
Other lunch	4.9	0.4	3.7	0.3	4.5	0.3
*p*-value		0.016		0.42		0.06
Added sugars (tsp. equiv.) ^2^						
School lunch	1.7	0.3	1.8	0.2	1.8	0.3
Other lunch	3.7	0.9	1.8	0.4	2.3	0.5
*p*-value		0.0001		0.97		0.47
Meal solid fats (g equiv.)						
School lunch	8.4	0.7	8.7	0.8	11.7	11.7
Other lunch	13.1	1.1	9.2	1.6	9.1	9.1
*p*-value		0.0002		0.84		0.18
Vegetables (cup equiv.) ^3^						
School lunch	0.28	0.02	0.25	0.06	0.19	0.04
Other lunch	0.35	0.03	0.33	0.07	0.35	0.06
*p*-value		0.025		0.40		0.01
Fruits (cup equiv.) ^4^						
School lunch	0.34	0.03	0.39	0.11	0.28	0.06
Other lunch	0.12	0.02	0.17	0.07	0.23	0.07
*p*-value		<0.0001		0.07		0.60
Grains (ounce equiv.)						
School lunch	1.8	0.1	1.9	0.2	2.5	0.2
Other lunch	2.7	0.2	2.1	0.3	2.5	0.2
*p*-value		0.002		0.52		0.80
Whole Grains (ounce equiv.)						
School lunch	0.08	0.02	0.15	0.07	0.07	0.03
Other lunch	0.13	0.06	0.07	0.03	0.24	0.04
*p*-value		0.39		0.32		<0.0001
Refined Grains (ounce equiv.)						
School lunch	1.77	0.09	1.71	0.16	2.39	0.22
Other lunch	2.58	0.18	2.02	0.32	2.29	0.15
*p*-value		0.002		0.385		0.630
Protein Foods (ounce equiv.) ^5^						
School lunch	1.3	0.1	0.9	0.1	1.4	0.2
Other lunch	2.0	0.2	1.2	0.2	1.5	0.3
*p*-value		0.002		0.14		0.91

Regression models with least-squared means presented, representing population intake during the lunch meal. Models are adjusted for age in years, sex, race, and energy intake. ^1^ PIR, Poverty-Income Ratio based on household size and survey cycle year. ^2^ Added sugars is calculated using sugar content of foods and ingredients defined by the USDA as added sugars. ^3^ Vegetables includes dark green, red, orange, starchy and other vegetables. ^4^ Fruits includes total intact fruit (whole or cut) and fruit juices used as sweeteners in foods, but excludes fruit juices consumed as beverages. ^5^ Protein foods includes meat, poultry, organ meat, cured meat, fish, eggs, nuts, seeds, and soy products.

## References

[1] Ogden C.L., Carroll M.D., Fryar C.D., Flegal K.M. (2015). Prevalence of Obesity Among Adults and Youth: United States, 2011–2014. NCHS Data Brief.

[2] USDA Food and Nutrition Service (FNS) (2016). National School Lunch Program (NSLP). http://www.fns.usda.gov/nslp/national-school-lunch-program-nslp.

[3] Woo Baidal J.A., Taveras E.M. (2014). Protecting progress against childhood obesity—The National School Lunch Program. N. Engl. J. Med..

[4] USDA, Food and Nutrition Service (2012). Nutrition Standards in the National School Lunch and School Breakfast Programs. Final Rule.

[5] US Department of Health and Human Services, Centers for Disease Control and Prevention, National Center for Health Statistics (2013). Healthy People 2010.

[6] US Department of Health and Human Services Healthy People 2020. http://www.healthypeople.gov/2010/hp2020/default.asp.

[7] Condon E., Drilea S., Lichtenstein C., Mabli J., Madden E., Niland K. (2015). Diet Quality of American School Children by National School Lunch Participation Status: Data from the National Health and Nutrition Examination Survey 2005–2010. http://www.fns.usda.gov/sites/default/files/ops/NHANES-NSLP05-10-Summary.pdf.

[8] Mancino L., Todd J.E., Guthrie J., Lin B.H. (2011). How Food Away From Home Affects Children’s Diet Quality.

[9] Crepinsek M.K., Gordon A.R., McKinney P.M., Condon E.M., Wilson A. (2009). Meals offered and served in US public schools: Do they meet nutrient standards?. J. Am. Diet. Assoc..

[10] Clark M.A., Fox M.K. (2009). Nutritional quality of the diets of US public school children and the role of the school meal programs. J. Am. Diet. Assoc..

[11] Caruso M.L., Cullen K.W. (2015). Quality and cost of student lunches brought from home. JAMA Pediatr..

[12] Ahluwalia N., Dwyer J., Terry A., Moshfegh A., Johnson C. (2016). Update on NHANES Dietary Data: Focus on Collection, Release, Analytical Considerations, and Uses to Inform Public Policy. Adv. Nutr..

[13] National Health and Nutrition Examination Survey. https://www.cdc.gov/nchs/nhanes/index.htm.

[14] U.S. Department of Agriculture, Agricultural Research Service, Beltsville Human Nutrition Research Center, Food Surveys Research Group, U.S. Department of Health and Human Services, Centers for Disease Control and Prevention, National Center for Health Statistics (2014). What We Eat in America NHANES 2013–2014.

[15] Bowman S., Clemens J.C., Thoerig R.C., Friday J.E., Shimizu M., Moshfegh A.J. (2013). Food Patterns Equivalents Database 2009-10: Methodology and User Guide.

[16] Vernarelli J.A., Mitchell D.C., Hartman T.J., Rolls B.J. (2011). Dietary energy density is associated with body weight status and vegetable intake in U.S. children. J. Nutr..

[17] Johnson L., Wilks D.C., Lindroos A.K., Jebb S.A. (2009). Reflections from a systematic review of dietary energy density and weight gain: Is the inclusion of drinks valid?. Obes. Rev..

[18] Briefel R.R., Wilson A., Gleason P.M. (2009). Consumption of low-nutrient, energy-dense foods and beverages at school, home, and other locations among school lunch participants and nonparticipants. J. Am. Diet. Assoc..

